# Large Scale Homing in Honeybees

**DOI:** 10.1371/journal.pone.0019669

**Published:** 2011-05-13

**Authors:** Mario Pahl, Hong Zhu, Jürgen Tautz, Shaowu Zhang

**Affiliations:** 1 BEEgroup, Biocentre, Würzburg University, Würzburg, Germany; 2 Centre of Excellence in Vision Science, Research School of Biology, The Australian National University, Canberra, Australia; Lund University, Sweden

## Abstract

Honeybee foragers frequently fly several kilometres to and from vital resources, and communicate those locations to their nest mates by a symbolic dance language. Research has shown that they achieve this feat by memorizing landmarks and the skyline panorama, using the sun and polarized skylight as compasses and by integrating their outbound flight paths. In order to investigate the capacity of the honeybees' homing abilities, we artificially displaced foragers to novel release spots at various distances up to 13 km in the four cardinal directions. Returning bees were individually registered by a radio frequency identification (RFID) system at the hive entrance. We found that homing rate, homing speed and the maximum homing distance depend on the release direction. Bees released in the east were more likely to find their way back home, and returned faster than bees released in any other direction, due to the familiarity of global landmarks seen from the hive. Our findings suggest that such large scale homing is facilitated by global landmarks acting as beacons, and possibly the entire skyline panorama.

## Introduction

Honeybee foragers have to provide a constant flow of nectar, pollen, water and propolis to the colony. The navigational information necessary for their frequent long distance flights is acquired from celestial and terrestrial cues. In order to keep track of the current position relative to the goal, forager bees employ several strategies. When first leaving the hive, young foragers perform systematic flight manoeuvres, backing away from the hive in a series of increasing arcs [1]. During those **orientation flights**, the animals memorize the hive itself, local landmarks surrounding the hive and global landmarks around the area [2], [3], [4]. When flying between nest and food source, the bee can then match the memorized cues with the actual visual environment [5]. The flight distance is estimated by **optic flow** experienced by the bee on the outbound route [6], [7]. When forced to fly in a non-beeline, i.e. around large obstacles like mountains, honeybees employ a **dead reckoning** system which constantly updates the distance and direction to the hive. Thus, in the waggle dance, the dancer communicates the straight line and distance to the resource, rather than the absolute distance flown around the obstacle [2]. Using direct light from the sun and polarized skylight detected by specialized ommatidia in the eye's dorsal rim area [8], the honeybee's **celestial compass** is able to measure angular movement relative to a reference direction, the solar meridian [9]. As a compass-backup for cloudy days, the skyline panorama is memorized together with the solar ephemeris function [10], [11]. En route to a goal, **familiar landmarks** can break down a trip into several segments to improve accuracy [12], and panoramic cues allow the recognition of landmark cues that, in turn, trigger local vectors [13]. These systems are flexibly applied to the task at hand. Chittka and colleagues have shown that when foraging by familiar landmarks, honeybees are able to suppress their path integration system, even when those landmarks are displaced. Alternatively, when forced to forage in a novel location without learnt landmarks, they use path integration without landmarks to navigate back to the hive [14].

Homing after displacement to unfamiliar regions has been investigated in various hymenopterans such as solitary sphecid wasps, *Cerceris tuberculata* [15], [16] and *Cerceris hortivaga* [17], social wasps, *Polistes gallicus* [18] and *Vespa orientalis* [19], solitary bees, *Dasypoda altercator* and *Osmia* sp.[20], [21], the social bees *Bombus terrestris* [22] and *Apis mellifera* [23], [24], [25], and several ant species (reviewed in [26]) for more than a century. Homing success in flying hymenopterans usually declines with increasing displacement distance, but the rate of decline is quite different between species. The maximum distance from which bees return after displacement varies widely from 200 m in *Pithitis smaragdula* [27] to 23 km in *Euplusia surinamensis* [28], [29], and is believed to be a good indicator for a species' maximum foraging range [30]. In studies on honeybees, the maximum homing distance ranges from 6 km [25] to 9.2 km [23]. To further investigate the honeybees' navigational abilities, we captured pollen foragers that had just returned to the hive, artificially displaced them in a black box to various destinations, and measured the time each bee took to come back home. Thus, we deprived the bees of any distance or directional celestial information about the release location in relation to the hive. The bees had to rely on knowledge they already had about the landscape.

Human observation can only be carried out reliably for a few hours at a time, which makes it difficult to gauge the behavior of large numbers of foragers over a long study period, such as days or weeks. It is precisely to overcome such difficulties that some researchers have turned to miniature signaling devices that can be attached to the thorax of individual bees, thereby allowing their behavior to be monitored automatically. One such technique involves the use of harmonic radar, with which the exact trajectories of individuals can be monitored over short periods of time, up to 1000 m from the radar device [29], [31]. We decided to use radio frequency identification (RFID) tags to be able to record the incoming and outgoing flights of many individual foragers at once, and over a time period of several days. While flight trajectories were not recorded, the small size of the RFID tags ensured undisturbed behavior of the bees, and no range limit in picking release sites. This was an improvement on previous techniques [23], [24], [25], because the exact return times and subsequent flight behavior of many individual bees could be measured, without the need of constant human observation. Even bees returning outside of normal observation hours and after several days in the field were recorded. Each tag was coded with an individual ID, which was logged by a receiver every time a tagged honeybee passed near it. Identification number, time and direction of movement were recorded by the receiver every time a forager returned home after an artificial displacement.

## Materials and Methods

### Experimental bees

The experimental *Apis mellifera ligustica* bees were housed in a two-frame observation hive containing approximately 3000 animals, connected to the outside via a perspex tunnel. The hive box was situated indoors in the Australian National University's native animal enclosure (35° 16′ 49.09″S, 149° 06′ 41.68″E, elevation 563 m). Each bee was tested only once.

### Experimental procedure

Pollen-carrying bees were captured upon return from a foraging trip at the hive entrance and briefly immobilized on ice, so that a RFID tag with known id number could be glued to each bee's thorax with shellac glue from a queen marking kit. Groups of 20 tagged bees were then kept in cages with *ad libitum* access to 50% sucrose solution. The cages were transported to the respective release sites in dark styrofoam containers so that the bees did not derive any directional information before the experiments began. The preparations were conducted in the morning, so that the experimental bees could be released in the early afternoon. At the respective release sites, the cages were opened at one side, and the bees were given 5 minutes to take off. The bees then spiralled upwards in wide circles until they were lost from view; homing trajectories could therefore not be determined. Animals which had not left the cage after 5 minutes were excluded from the experiment. Approximately two hours passed between the bees' capture and release. Upon return to the hive, the bees' identity and homing time were recorded by the RFID receivers at the hive entrance.

### RFID system

Each bee was equipped with a RFID tag on the thorax (2.0×1.6 mm, 2.4 mg, Microsensys mic3-TAG 64-D). All tags carried a unique 64 bit number, which allowed us to individually track the experimental bees' flight behavior. Two RFID receivers (Microsensys 2k6 HEAD) attached to the hive tunnel recorded each in- and outbound flight of the tagged bees.

### Landscape of the experimental area

**Figure 1:**
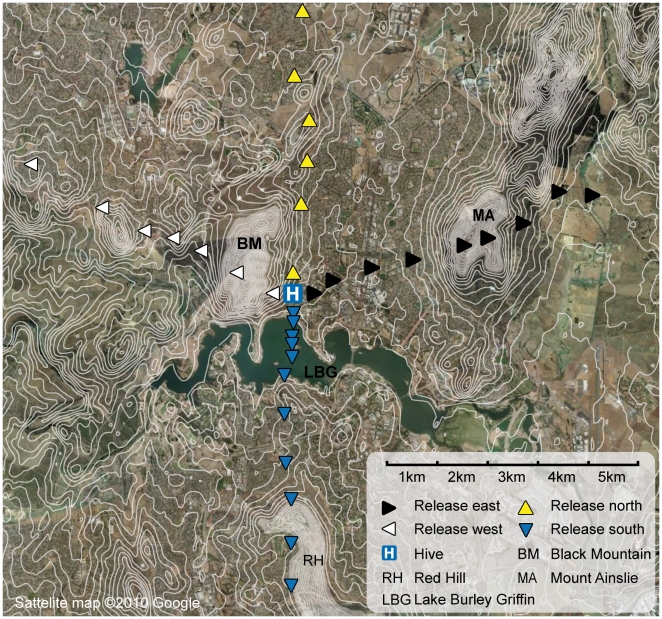
Map of the experimental area. 20 bees were released at each marked spot. White lines show terrain contour, and white areas denote hills blocking the direct view to the vicinity of the hive. Up, down, left and right-pointing triangles indicate releases in the north, south, west and east, respectively.

**Figure 2:**
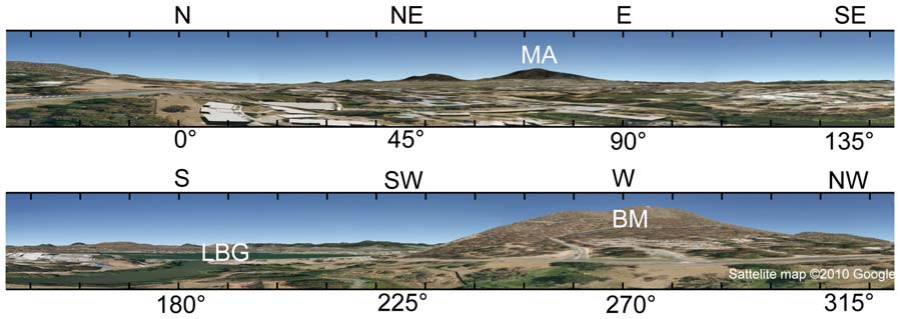
Panoramic view of the experimental area, as seen from the hive. Buildings and trees are flattened; the viewpoint elevation is 15 m. Note the distinctive shapes of Black Mountain (BM) in the west and Mount Ainslie (MA) in the east. Lake Burley Griffin (LBG) lies south of the hive.

The experimental area is shown in the satellite map in Figure 1, and the surrounding panorama as seen from the hive is shown in Figure 2. We released groups of bees in the four cardinal directions in various distances from the hive. In the **eastern direction**, the bees were released in rural areas (up to 3300 m distance), on top of and behind the 830 m high Mount Ainslie (MA, 4400 m to 7800 m distant), and further away (up to 13000 m) behind MA. Black Mountain (BM, elevation 810 m) was visible from the rural areas and from the top of MA (4400 m away), but not from the release spots further away, where MA blocked the direct line of sight. We chose a line of release spots slightly north easterly from the hive, in order to use the peak of MA as a visual barrier for the bees at the distant release spots behind the mountain. The release spots in the **western direction** were chosen in a way similar to the eastern ones, i.e. to have the large visual barrier of BM between the hive and the distant release spots. Behind the 1400 m spot on top of BM, the mountain was still visible from all release spots, but from a different angle than the one the bees were used to. MA was not visible from behind BM. In the **northern direction**, the bees were released in rural areas at a maximum distance of 7000 m from the hive. BM and MA were visible from all spots, although from an unfamiliar angle. In the **south**, the line of release spots crossed Lake Burley-Griffin (LBG). Bees homing from 800 m to 1500 m distance were released from a boat. BM and MA were visible from all releases up to the 5000 m spot on top of Red Hill (RH), but not from the spots behind RH at 6 and 7 km.

### Weather

Experiments were conducted solely in fine weather conditions. On all experimental days, the average temperature was between 25 and 35°C, the sky clear or partly overcast with a visibility of at least 10 km. The wind usually blew from the north-east with an average speed of 15 km/h.

### Data analysis

The homing rate for each release spot was determined as the number of returning bees divided by the number of released bees. The time between take-off at the release site and the first reading of each bee at the hive was determined to be the individual homing time. Median homing time was calculated for each release across all bees returning on the same day. Bees returning on the next day were excluded from the homing time analysis, but not from the homing rate analysis. Homing speed was calculated for each bee returning on the same day, as the release distance divided by the individual homing time. This measure does not represent flight speed, as it includes searching, resting and refueling on the way.

## Results

### Homing rate and homing time

**Figure 3:**
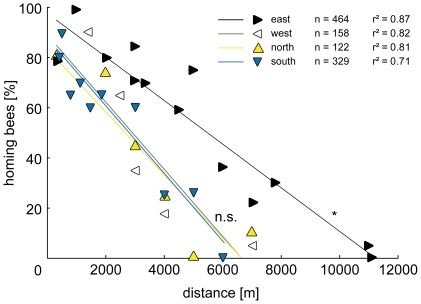
Homing rate in the four cardinal directions. Beginning at 80–100% close to the hive, the proportion of returning bees declines to 0% at around 6 km in the west, north and south, and at 11 km in the east. Homing rate from the eastern direction is consistently higher than from north, west and south (Comparison of slopes, p<0.033. Each point is based on 20 bees). Up, down, left and right-pointing triangles indicate releases in the north, south, west and east, respectively.

**Figure 4:**
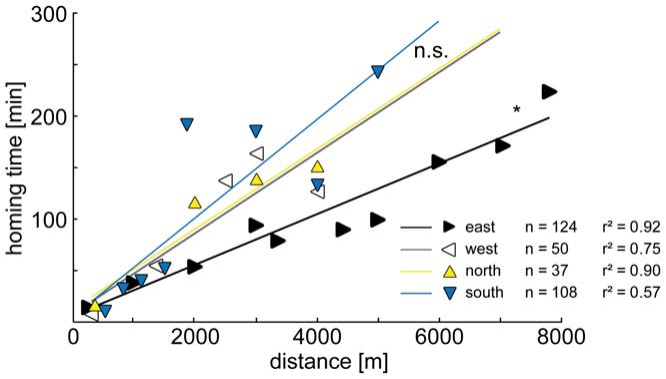
Homing time in the four cardinal directions. Bees returning from the east take less time than bees returning from the west, north and south (comparison of elevations and intercepts, p = 0.011). Up, down, left and right-pointing triangles indicate releases in the north, south, west and east, respectively.

In all four directions, there was a negative linear relationship between homing rate and distance, and a positive relationship between homing time and distance (Figs. 3 and 4). There was no significant deviation from linearity in homing rate (Runs test, p_(east)_ = 0.825, r^2^_(east)_ = 0.877; p_(west)_ = 0.700, r^2^_(west)_ = 0.824; p_(north)_ = 0.800, r^2^_(north)_ = 0.809; p_(south)_ = 0.955, r^2^_(south)_ = 0.707) or homing time (Runs test, p_(east)_ = 0.788, r^2^_(east)_ = 0.933; p_(west)_ = 0.500, r^2^_(west)_ = 0.754; p_(north)_ = 0.667, r^2^_(north)_ = 0.899; p_(south)_ = 0.222, r^2^_(south)_ = 0.569) in any of the four directions. Consequently, the data were analyzed by linear regression.

In the **east**, the maximum homing rate was recorded at 1000 m, from where all bees returned. The maximum homing distance was 11000 m (Fig. 3), and the maximum homing speed of 50.51±9.07 m·min^−1^ was recorded from the 5000 m spot (Table 1). In the **western** direction, a maximum of 90% returned from the 1400 m release on top of BM, probably due to the exceptional view of the surrounding area from the mountain peak. The maximum homing distance was 7000 m (Fig. 3), and the maximum homing speed of 31.01±12.35 m·min^−1^ was reached at 4000 m (Table 1). **North** of the hive, the highest homing rate was reached at 300 m, where 78.9% of the bees returned at a speed of 33.75±12.43 m·min^−1^ (Table 1). The maximum homing distance was 7000 m (Fig. 3). In the **south**, the highest percentage of bees returned from the 520 m release at the lake shore (89.5%, Fig. 3). The fastest homing flight from south was recorded at the 520 m spot, where the bees returned at an average pace of 65.00±11.66 m·min^−1^ (Table 1).

**Table 1:** Homing speed and number of released bees.

	East	West	North	South
Median homing speed [m·min^−1^]	36.90±3.26	24.27±3.69	23.53±5.01	26.43±3.89
Maximum homing speed	50.51±9.07	31.01±12.35	33.75±12.43	65.00±11.66
at distance	5000 m	4000 m	300 m	520 m
n_(returned in 24h)_	124	50	37	108
n_(returned)_	154	64	48	128
n_nreleased)_	464	158	122	329

This table shows the median homing speed in the four release directions and the highest homing speed at the respective release distance. The number of bees returning inside the 24 h after release, the number of bees that returned at any time after release and the total number of released bees are noted.

Figure 3 shows that the best-fit lines for the **homing rates** from west, north and south do not differ significantly from each other (linear regression; slopes: f = 0.012, DFn = 2, DFd = 18, p = 0.988; elevations & intercepts: f = 0.059, DFn = 2, DFd = 20, p = 0.943). Thus, the data were pooled and compared to the eastern direction. There was a significant difference between the best-fit lines for the homing rates from the east and the pooled data from west, north and south (linear regression; slopes: f = 4.958, DFn = 1, DFd = 32, p = 0.033).

Similarly, Figure 4 shows that the best-fit lines for the **homing times** from west, north and south are not significantly different from each other (linear regression; slopes: f = 0.014, DFn = 2, DFd = 13, p = 0.986; elevations and intercepts: f = 0.172, DFn = 2, DFd = 15, p = 0.843). Accordingly, the data were pooled and compared to the eastern direction. Linear regression showed a significant difference between the elevations and intercepts of the best-fit lines (f = 7.489, DFn = 1, DFd = 27, p = 0.011), but not between the slopes (f = 1.996, DFn = 1, DFd = 26, p = 0.170).

**Figure 5:**
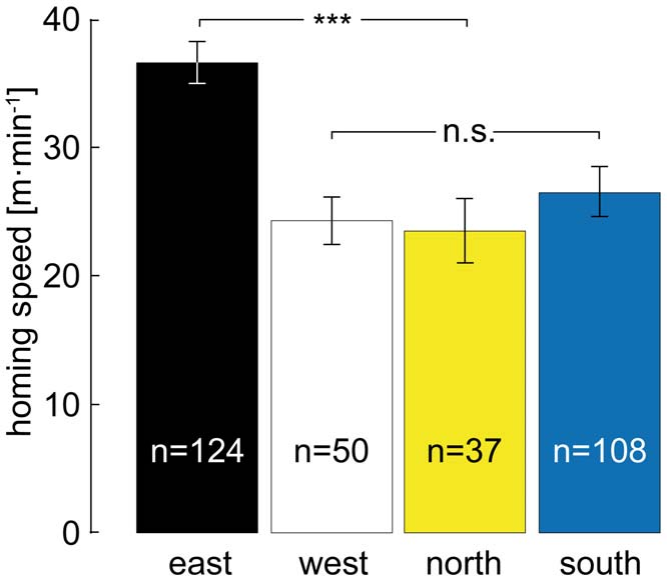
Homing speed. Bees homing from the eastern direction return to the hive sooner than bees from the west, north and south. *** Denotes p<0.001; n.s. = not significant. Error bars show SEM.

The average **homing speed** of bees returning from the west, north and south was around 25 m·min^−1^, about 10 m·min^−1^ slower than the homing speed from the east (Fig. 5). The speeds from the west, north and south did not differ from each other (ANOVA, p = 0.697). Consequently, they were pooled and compared to the homing speed from the east, which was significantly higher than the speeds of bees returning from the west, north and south (t = 14.379, df = 317, p<0.001).

**Figure 6:**
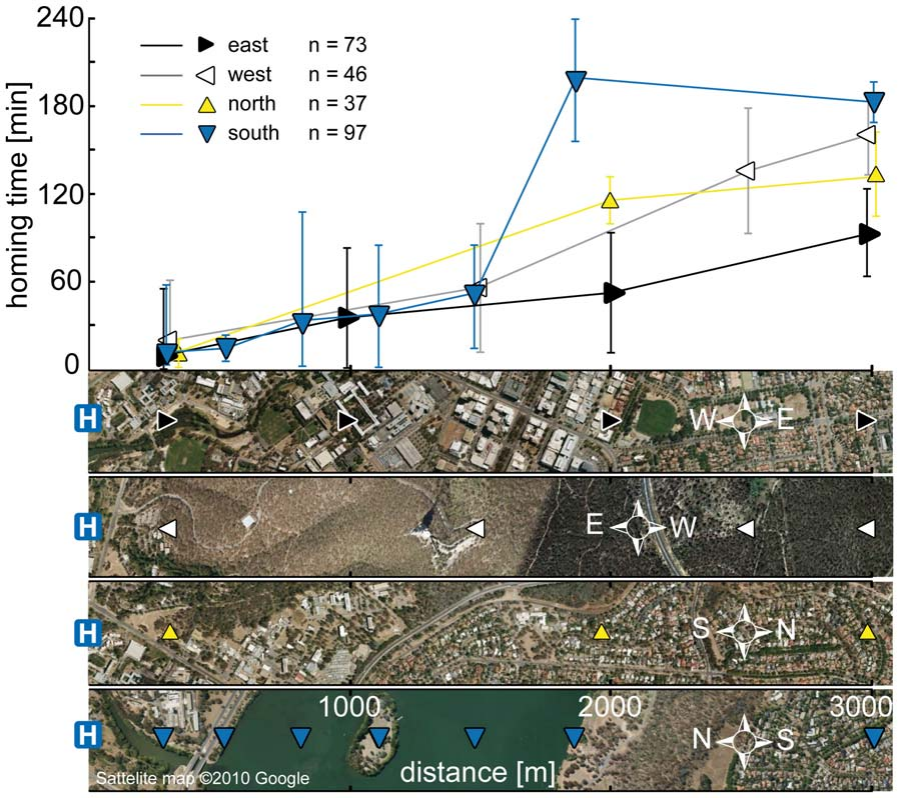
Homing time over land vs. water. The graph compares homing times from up to 3000 m in four directions. Map strips show release spots over different terrain: urban areas in the east, forests west of the hive, urban areas north and the lake south of the hive. 20 bees were released at each spot, and homing times were calculated from bees returning on the day of release. Error bars show SD.

In the southern direction, some bees were released over water. Fig. 6 shows the terrain of the release spots up to 3000 m from the hive, and compares the southern homing times to those measured for the other directions. The homing times for close distances up to 1500 m were similar in all directions. When released on the opposite side of the lake, however, homing times increase drastically from an average of 52±12 min at 1480 m to 193±25 min at 1870 m; an almost fourfold increase in time, while the distance is only 400 m further.

## Discussion

Many of the honeybees found their way back home even after blind displacement to unfamiliar areas, some of them from up to 11 km. Our RFID setup monitored a large number of individual bees around the clock for many days. It produced precise measurements by recording the exact arrival time of each animal, and ensured that no late arrivals were missed.

Since the bees could not perceive the direction of movement during the displacement, compass information alone (be it from the sun, the polarization pattern in the sky, magnetic inclination or polarity) could not have guided them. Catching experimental forager bees upon return to the hive ensured that the bees' path integrator was set back to 0, and thus had no influence on the bees' homing direction. Local landmarks around the hive were not visible from release spots further than 500 m away, and even global landmarks like BM were not always visible (on the release sites further than 4000 m in the east).

The typical honeybee foraging range depends on the abundance of food, water and propolis around the hive. Most resources are collected within a 600–800 m radius, although distances of 2 km are still common, and bees may even travel 5 km in some situations [23], [32]. Only in extreme experimental conditions of food and water deprivation do bees venture to maximum distances of 13 km [33]. However, the experimental hive was situated only 300 m from the Canberra National Botanical Gardens, a year-round source for pollen, nectar and propolis. Thus, it is unlikely, but not impossible, that the bees knew the areas beyond the lake, behind BM and beyond MA. How could they find the way back? When bees leave the hive for the first time, they perform orientation flights, a series of steadily increasing arcs in which they familiarize themselves with the surrounding area (reviewed by [1]). Those trips are essential for successful homing; bees artificially displaced before the first orientation flight have trouble finding their way back home even from a 50 m distance [25]. The hive itself, the surrounding local landmarks and global landmarks from the horizon panorama are memorized to make sure they find their way back home after the first foraging trip. Bees learn the sun's pattern of movement in relation to the entire landscape panorama around their nests, enabling them to extract the solar ephemeris function even on cloudy days from the surrounding skyline [11], [34]. Ants have recently been shown to use the panoramic skyline to determine the homewards direction after artificial displacement [35]. Despite the difference in scale, it is likely that bees can use similar visual cues for homing after displacement.

Most foragers in our study had no trouble flying back from the close release spots in a 1500 m radius around the hive. On this small scale, familiar local features can guide bees towards frequent foraging routes or directly to the hive [36]. Especially high homing rates were recorded from the release site 1400 m west on top of BM, from where 90% of the bees returned due to an exceptionally good view of the area surrounding the hive, and the 1000 m eastern release, from where all bees returned. Earlier studies have looked at homing from different directions only in close distances up to 2000 m. They found no difference in homing success or homing time between different directions close to the hive [24], [25], consistent with our results.

On a medium scale, up to about 4000 m, the homing rates from the eastern releases are much higher than from the other directions (Fig. 3). Bees homing from the east spend less time finding their way home than bees homing from the other directions (Fig. 4). The panorama between the two mountains BM and MA is familiar to the bees, since the orientation flights are performed in this area. Thus, BM could act as a beacon, guiding bees towards the hive. Bees familiar with the area could also have vector memories associated with global landmarks like BM and MA. Retrieved in the right panoramic context, memories encoding distance and direction to the nest could guide the bees home or to the next familiar path segment [13]. The directional component of the vector could either be provided by the polarization compass, or the panorama itself. Another possible mechanism is the use of the entire skyline panorama [35], [37]. The bees could home in towards the hive by minimizing differences between the stored, familiar panorama around the hive, and the actual surrounding view, e.g. flying away from MA westwards to BM [38], [39]. The distinctive shape of BM as seen from the hive (Fig. 2) could also be directly used as a landmark beacon. Southwick and Buchmann (1995) released bees at a 3900 m distance from their hive in the four cardinal directions. In a flat, featureless experimental area, where the maximum homing distance was 5600 m, they found no difference between the homing rates in the four directions, probably due to the missing panoramic cues. In a mountainous experimental area, where bees returned from up to 9200 m, they studied only one release direction, south-east along a mountain ridge. In this area, with a prominent panoramic skyline around the hive, they might have found significant differences between the release directions as well.

On the larger scale, further than 7000 m distant, only bees from the east successfully returned home. BM is not visible from the release spots further than 4400 m in the east, since MA is blocking the view. Even so, 30–40% of the bees returned from the releases behind MA. Mechanisms similar to those operating in the medium scale could be at work here: by flying towards a mountain in the west, the released bees would fly to MA first and then continue towards BM, where familiar local features eventually take over and guide the bees to the hive. This would also explain the lower homing rates from the other directions: flying west towards the next mountain from those release sites would only take the bees further away from the hive.

The flight time for the homing trip increased with distance. Flying at a pace of 15 km/h, even the most distant release spots were easily reachable after a 60 minute flight. However, the homing times were always much higher than expected at the usual travel speed of a bee. Sometimes, e.g. from the 11000 m spot in the east, it took several days for a bee to return to the hive. Homing times, e.g. from the 3000 m spots, varied between 78 min from the east and 280 min from the south. This indicates that the time spent searching for the correct heading is much longer than the actual travel time, and significantly different for each direction. The actual distances travelled by the bees, were they constantly flying at 15 km/h, could be as much as 19.5 km from the east and 70 km from the south. To cover such distances, the bees would have to drink nectar to refuel on the way, since a crop load of 20 µl 1.3 M sugar solution will keep a bee flying for just about 25 min, or 7 km [40].

Bees homing from the southern release spots on the lake took as much time as those homing from equal distances from the other directions (Fig. 6). When released from the opposite shore, however, homing speed decreased from 28.47±6.39 m*min^−1^ at 1480 m (last release on the lake) to 9.69±3.14 m*min^−1^ at 1870 m (first release on opposite shore). It is unlikely that the bees were just flying slower from the release on the southern lake shore, since homing speed is no measure of flight speed, but includes searching, resting and refueling time. The two release sites were only 400 m apart, have the same elevation and share a similar view of the surrounding area. Moreover, a comparable percentage of bees found the way back to the hive (60% from 1480 m and 65% from 1870 m), indicating that the bees did not have more trouble locating the hive from the opposite lake shore. Since bees are generally hesitant to fly over water [2], [41], they most likely chose the detour over land along the shore from the 1870 m spot, and took the direct route from the release on the lake.
